# Elk and Deer Habituate to Stationary Deterrents in an Agricultural Landscape

**DOI:** 10.1002/ece3.71752

**Published:** 2025-07-10

**Authors:** Kate L. Rutherford, Colleen Cassady St. Clair, Darcy R. Visscher

**Affiliations:** ^1^ Department of Biological Sciences University of Alberta Edmonton Alberta Canada; ^2^ Department of Biology King's University Edmonton Alberta Canada

**Keywords:** acoustic deterrent, anti‐predator behavior, habituation, human–wildlife interactions, multimodal stimuli, wildlife management

## Abstract

Deterrents that are designed to emulate humans or natural predators are increasingly applied to manage the behaviors and distribution of conflict‐prone species, but the efficacy of these tools is frequently challenged by the process of habituation. In this study, we investigated the responses of Roosevelt elk (*Cervus canadensis roosevelti*) and black‐tailed deer ( *Odocoileus hemionus columbianus* ) to playbacks of unimodal (acoustic) and multimodal (acoustic and visual) stimuli on crop fields in the Cowichan Valley, British Columbia, Canada. We contrasted behavioral responses to acoustic recordings of (i) human voices (shouting and talking), (ii) natural predator vocalizations (wolf and cougar), (iii) dog barks, and (iv) local bird vocalizations (control) and tested the effect of flashing LED lights using alternating audio‐light and audio‐only treatments at the same sites over two 3‐week periods. We found that multimodal stimuli increased the likelihood of fleeing by 4.7 in elk and 1.8 in deer but did not affect the time spent in alert postures. Among acoustic treatments, playbacks of human shouts tended to elicit greater flight responses than humans talking, natural predators, dogs, and bird sounds. Both species showed evidence of habituation to the deterrents as the 6‐week experiment progressed, but elk responses declined more rapidly than deer, and rates of habituation for both species were slower when deterrents included flashing lights. For deer, alert responses declined more rapidly at sites surrounded by more houses and closer to highways. Together, our results indicate that recordings of humans shouting provided the most salient acoustic deterrent for these ungulates and that acoustic deterrents were enhanced with lights, but habituation to our stationary deterrents occurred rapidly, especially in proximity to human activity.

## Graphical abstract

This study investigated the responses of Roosevelt elk (*Cervus canadensis roosevelti*) and black‐tailed deer (*Odocoileus hemionus columbianus*) to acoustic and visual deterrents on crop fields in the Cowichan Valley, British Columbia. Both species were most likely to flee and exhibit alertness in response to playbacks of human vocalizations, while combining audio and light increased flight responses but not alert time. Additionally, habituation occurred rapidly, especially for elk and at sites in proximity to human activity.

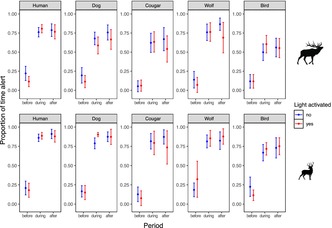

## 1 Introduction

Fear in animals, defined as the perception of risk in a given situation, contributes to the behavioral responses of individuals with potential consequences for the dynamics and distribution of populations (Brown et al. 1999; Stankowich and Blumstein 2005). Fear‐induced behavior appears to be especially important in prey species as an adaptation to mitigate the risk associated with predation (Lima 1998; Clinchy et al. 2013). Within species, additional variation in fearfulness may occur among individuals owing to differences in body condition (Clark 1994), personality (Found and St. Clair 2019), environmental conditions (Owen et al. 2017), and habitat characteristics (Epperly et al. 2021). Understanding these sources of variation among and within species could increase the potential to use fear as a tool to achieve objectives for wildlife management and conservation (Cromsigt et al. 2013; Berger‐Tal et al. 2016; Gaynor et al. 2021).

One of the prevalent applications of fearfulness in wildlife is the use of deterrents that are intended to dissuade animals from using areas or resources that are valued by people (Marsh et al. 1992; Smith et al. 2000; Conover 2002; King et al. 2007; Schakner and Blumstein 2013; Babińska‐Werka et al. 2015; Widén et al. 2022) or protect animals from human‐caused hazards such as oil spills (Ronconi et al. 2004) or industrial sites (Stevens et al. 2000; Ronconi and St. Clair 2006). In both contexts, proponents often use deterrents to reduce mortality of wildlife, whether through incidental actions by humans or by reducing the need for lethal management of problem animals (Miller et al. 2016). Typically, deterrents use a variety of acoustic, olfactory, and visual cues to induce fear (anti‐predator responses) or create perceived foraging costs (i.e., ‘olfactory misinformation’; Price et al. 2022; Parker et al. 2023) to manipulate the behavior and distribution of wildlife (Cromsigt et al. 2013; Gaynor et al. 2021). For audio deterrents, the fear of human voices appears to be especially influential, often eliciting stronger anti‐predator responses than natural predator vocalizations in a variety of terrestrial vertebrates (Smith et al. 2017; Suraci et al. 2019; Crawford et al. 2022). In addition to acoustic cues, visual stimuli such as flashing lights can function as aversive signals by increasing the salience or unpredictability of a perceived threat. Light‐based deterrents may trigger startle or vigilance responses in prey, particularly in low‐light conditions, by mimicking predator eyeshine or human presence (Koehler et al. 1990). Unfortunately, the efficacy of deterrents for reducing human‐wildlife conflict is frequently challenged by habituation, a behavioral process in which animals learn to ignore stimuli that have no fitness consequences (Groves and Thompson 1970; Blumstein 2016).

Decades of work on the use of deterrents for common applications, such as agriculture and aquaculture, have shown that habituation is typically slower when the stimuli simulate realistic sensory cues of natural predators (Kloppers et al. 2005; Enos et al. 2021) and are activated in response to animal arrival or movement (Belant et al. 1996; Stevens et al. 2000; Beringer et al. 2003; Ronconi and St. Clair 2006; Chilvers 2024). Studies on diverse taxa have demonstrated that presenting multimodal predator or conspecific alarm cues can increase antipredator effort relative to a single‐component stimulus (Smith and Belk 2001; Roberts et al. 2007; Partan et al. 2009). Additionally, several authors suggest that multimodal stimuli can delay habituation (Conover 2002; Elmer et al. 2021), although there have been few empirical investigations of this effect (Lecker et al. 2015). Animals may exhibit greater attention to multimodal stimuli because it increases their certainty about the associating threats (Munoz and Blumstein 2012). Habituation to an aversive stimulus, including a deterrent, can also be affected by the time between exposures, where irregularity and infrequency reduce the likelihood of habituation (Blumstein 2016; Zanette and Clinchy 2020). Additional features that affect the rate of habituation might include various functional traits of predator and prey, habitat features, and the degree of human disturbance on the landscape (Blumstein 2016; Hansen and Aanes 2015). Despite the wealth of literature on animal responses to aversive stimuli, many studies do not report specifically on rates of habituation (Ramirez et al. 2024) and there is an urgent need for more information, particularly in contexts and spatial scales that reflect actual sources of human‐wildlife conflict.

Ungulate depredation of crop fields is a prevalent and perennial source of human‐wildlife conflict throughout the world (Nyhus 2016; Seoraj‐Pillai and Pillay 2016; Gemeda and Meles 2018), which causes economic loss and lessens tolerance for wildlife (Can‐Hernández et al. 2019). Traditional solutions to mitigate this problem often focus on removing animals, such as through lethal control, or restricting access with exclusion fencing (Walter et al. 2010). However, lethal management faces growing public opposition, while exclusion fencing is expensive and sometimes impractical, so alternative approaches are needed (Vercauteren et al. 2006). On some spatial scales, this conflict may be mitigated with deterrents consisting of audio recordings, which have been used to address crop damage by ungulates in Sweden (Widén et al. 2022) and elephants ( *Elephas maximus* ) in India (Thuppil and Coss 2016). These systems typically employ remote cameras that trigger an aversive acoustic stimulus when animals approach, creating a temporally unpredictable but spatially predictable risk cue, thereby lessening the rate of habituation (Lima and Bednekoff 1999). Maximizing the efficacy of these new tools requires more information about how animals respond to different cue types over immediate and long‐term time periods and among different spatial contexts (Gaynor et al. 2021; Ramirez et al. 2024).

The goal of this research was to determine how ungulates respond to audio‐visual playback devices on crop fields over a 6‐week period in the Cowichan Valley region of Vancouver Island, British Columbia, Canada. There, intensive agriculture, growing urban areas, and a major highway co‐occur with the presence of two native ungulates: Roosevelt elk (*Cervus canadensis roosevelti*) and black‐tailed deer ( *Odocoileus hemionus columbianus* ). The local abundance of elk has caused increasing concern among some farmers in this region because elk depredate crops and damage fences (Bush 2024). We used camera‐triggered audio deterrents fitted with LED lights to (1) compare responses by both species to acoustic stimuli of various types, (2) determine whether acoustic stimuli were more effective when paired with LED lights, and (3) measure rates of apparent habituation via changes in responses over time among sites with differing levels of human activity. Based on the literature presented above, we predicted that elk and deer would flee more frequently and express greater alertness when exposed to acoustic stimuli that mimicked predators, when acoustic stimuli were paired with lights, and at sites with less human activity.

## 2 Materials and Methods

### 2.1 Study Area

**FIGURE 1:**
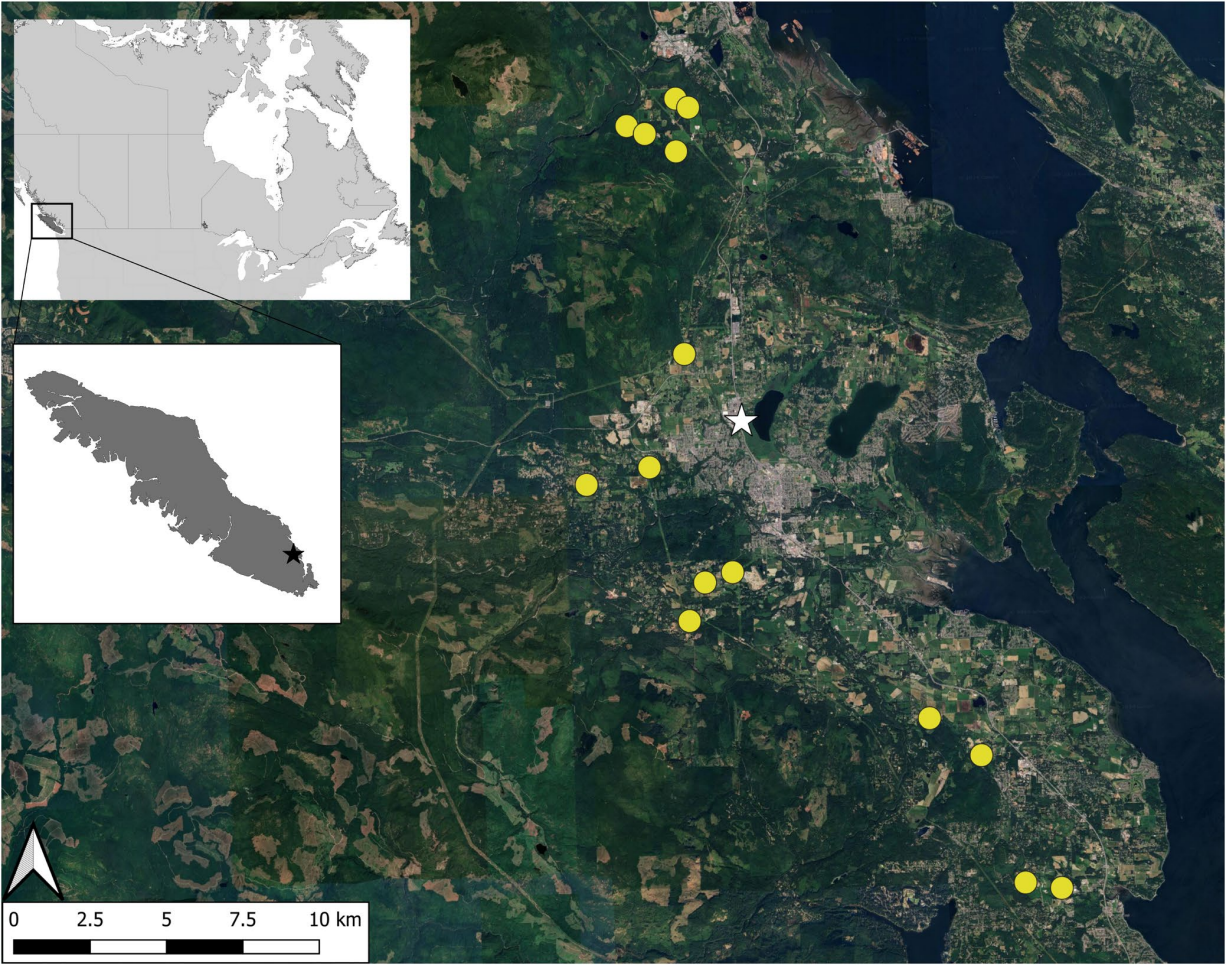
Locations of 15 motion‐activated deterrents (yellow circles) distributed on crop fields near the town of Duncan on Vancouver Island, British Columbia, Canada, from August 4th to September 17th 2023.

The Cowichan Valley Regional District is situated in the southeastern area of Vancouver Island (Figure 1) and we focused our study in the area near the town of Duncan. Our study region includes the municipality of North Cowichan, as well as two electoral areas, with population densities of 163.7, 31.6, and 29.4 people per square kilometer, respectively (Statistics Canada 2024). This area is made up of a mosaic of temperate rainforests, agriculture, and urban development. The mild maritime climate supports diverse agriculture consisting of crop fields (mostly hay and corn), orchards, vineyards, and farms, primarily dairy operations.

Populations of Roosevelt elk and black‐tailed deer are managed through annual regulated hunting, which occurs between September and December. The elk harvest is tightly regulated through limited entry hunting, wherein hunting authorizations are awarded based on a lottery system with approximately 15,000 annual applications for 300 draws province wide (Wilson 2015), which acknowledges the historic scarcity of this species (Spalding 1992). In 2022, elk tags were distributed to 211 resident hunters and 23 non‐resident hunters on Vancouver Island, 34 of which were for the Cowichan Valley management unit, which is designated for archery only (British Columbia Wildlife 2024). First Nations groups also receive tags to harvest elk through the limited entry system within the regulated hunting season. In contrast, abundant black‐tailed deer are hunted annually during an open season (September to December) by licensed hunters and year‐round by First Nations hunters. Potential predators for ungulates in the region include gray wolves ( *Canis lupus* ), cougars ( *Puma concolor* ) and black bears ( *Ursus americanus* ).

### 2.2 Experimental Design

**FIGURE 2:**
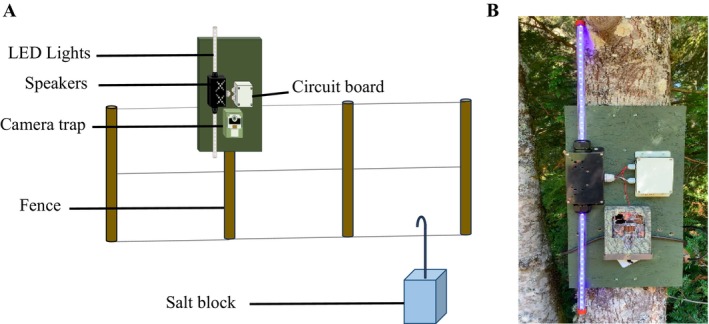
Diagram of BoomBox‐Disco deployment. (A) The BoomBox‐Disco components (speakers, LED lights, camera trap, and circuit board enclosure) were attached to painted green plywood and mounted to a tree or fence post at the edge of a crop field. A 20 kg salt block was staked 4–12 m in front of the camera as an attractant. (B) BoomBox‐Disco deployment in the field with LED lights flashing.

We conducted an experiment for 45 days (from August 4 to September 17, 2023) on 15 crop fields where we had previously detected elk presence with remote cameras and found activity to be highest during these months. On the perimeter of each crop field, we installed an Automated Behavioral Response (ABR) system called the ‘BoomBox‐Disco’. This ABR system functions by integrating a motion‐activated camera with a playback speaker unit and two LED light strips that generate sounds and flashing light when the camera's motion sensor is triggered (www.freaklabs.org). An earlier version of this device (the ‘BoomBox’) lacked the LED attachment, but has been used in field studies to measure the behavioral responses of herbivores, mesocarnivores, and primates to predator vocalizations (Palmer et al. 2022). To protect the hardware in the field, the circuit board and speakers were enclosed in a plastic housing. We created holes in the speaker box to increase audio transmission. We enclosed the LED light strips in two clear plexiglass tubes, which were attached vertically to the top and bottom of the speaker box. Each unit was connected to a camera trap (Browning Spec Ops Edge BTC‐8E). The four components (circuit board, speaker box, LED lights, and camera trap) were attached to painted green plywood, which was mounted to a tree or fence post at the edge of a crop field (Figure 2).

The cameras recorded 20 s videos when triggered (following Crawford et al. 2022), which were separated into three time periods: an 8 s period before the stimulus was displayed (silent “control” period), a 6 s period during which the stimulus was displayed, and a 6 s silent period after the stimulus was displayed. To determine how ungulates responded to the addition of flashing light, each site was randomly assigned to an audio‐light treatment (*n* = 8) or an audio‐only treatment (*n* = 7) for the first half of the study (3 weeks) and then switched to the opposite treatment for the second half of the study. We placed a 20 kg salt block in front of each camera (range 4–12 m) to attract animals to the focal range of cameras.

### 2.3 Acoustic Stimuli

We contrasted behavioral responses of elk and deer to four stimuli types: (1) human voices, (2) dog barks, (3) natural predator (wolf and cougar) vocalizations, and (4) bird vocalizations, which we presumed to be less threatening than the other stimuli (similar to Epperly et al. 2021; Crawford et al. 2022; Bhardwaj et al. 2022). To reduce habituation to the same audio clip, we used six exemplars of each of the four acoustic treatments. The human treatment consisted of men and women talking in a conversational tone (*n* = 3) and shouting (*n* = 3) in English, the dominant local language. Natural predator vocalizations consisted of cougar screams (*n* = 3) and wolf howls (*n* = 3). Dog barks were aggressive and included recordings of multiple breeds (*n* = 6). The bird treatment consisted of robin songs (*n* = 2), crow calls (*n* = 2), and nighthawk calls (*n* = 2). Most audio clips featured a single individual vocalizing (*n* = 20), while a few included vocalizations from multiple individuals (*n* = 4). The deterrent device was programmed to play the audio clips (*n* = 24) in a random order once triggered. Additionally, we programmed a 1‐min delay between trigger events to reduce repeated exposure to the stimuli. To increase consistency in sound quality and power, all sounds were edited in Audacity (www.audacityteam.org) to achieve sound power of 80 ± 5 dB, as measured with a decibel meter (TopTes TS‐501B), 1 m from the device. The volume of playbacks was not significantly different between audio treatments (*F*_3,20_ = 1.28, *p* = 0.31).

### 2.4 Behavior Analysis

We used an electronic ethogram to record the timing and duration of behaviors from camera trap videos using the open‐access software “BORIS” (Friard and Gamba 2016; Table 1). We defined a focal individual as the animal closest to the salt block when the stimulus began (at 8 s) and recorded its behaviors in each event. For each focal individual, we recorded the species, sex, age class (adult, juvenile, unknown), and whether the focal individual was the same focal individual from the previous event (yes, no, unknown). For each event, we recorded the site, date, time, trial day, acoustic stimulus played, whether the LED lights were activated, whether the focal individual left the field of view before playback, and the group size (number of individuals of the focal species counted in the event; Table 2). To determine when each event took place within the diel period (day or night), we compared the time of the event to the start and end times of local civil twilight. Daily sunrise and sunset tables were calculated for the nearest town using an on‐line calculator (www.nrc‐cnrc.gc.ca/eng/services/sunrise/).

**TABLE 1:** Ethogram coded in BORIS used to quantify behaviors of elk and deer on agriculture fields in the Cowichan Valley, BC.

Behavior	Description
Running	Moving at a fast pace, seemingly disturbed by something
Vigilant	Displaying clear alert behaviors (ears up and looking in a few directions) and observant of surroundings
Walking	Walking at a slow pace, seemingly not stressed or disturbed
Standing	Standing on all four hooves, seemingly undisturbed
Feeding	Browsing/grazing on vegetation or licking the salt block
Investigating	Investigating the camera or salt block
Out of frame	Animal is no longer visible in the field of view

*Note:* Behavior definitions are adapted from Bhardwaj et al. (2022).

**TABLE 2:** Response and explanatory variables used in analyses to predict elk and deer responses to deterrents on crop fields in the Cowichan Valley, BC.

Variable	Description
Response variables	
Fled	Binary variable quantifying if elk and deer fled the site during the video. Yes = 1, No = 0
Alert	The time elk and deer spent alert (vigilant, running, out of frame) proportional to the duration of the observation period (range 0–1)
Explanatory variables	
Audio	Factor for each acoustic stimulus: human (shout, talk), dog, natural predator (wolf, cougar), and bird control (robin, nighthawk, crow)
Lights	Factor with two levels: on or off
Trial day	Trial day of the experiment (range 1–45)
Sex age	Factor with four levels: adult female, adult male, juvenile, unknown adult
Group size	Number of individuals of the same species counted in the event
Diel period	Factor with two levels: day or night (whether the event occurred during daytime or nighttime based on daily local civil twilight start and end times)
Random effect	
Site	Factor of site ID 1–15

### 2.5 Statistical Analyses

#### 2.5.1 Probability of Fleeing and Proportion of Time Alert

Before exploring differences among audio stimuli types, we tested whether the probability of fleeing or being alert differed in response to stimuli within the groups of 3 birds, 2 natural predators, and 2 types of human sounds (Table S2). We found no differences in either response variable among bird sounds for both elk or deer (*p* ≥ 0.36 for all comparisons), but elk were more likely to flee from human shouts than talking (*χ* ^2^(1) = 6.17, *p* = 0.013) and were more often alert in response to wolves than cougars (*F*_(1,247)_ = 5.98, *p* = 0.015; for all other comparisons, *p* ≥ 0.14; Table S2). For consistency in subsequent analyses, we separated the human stimuli treatment into a “human‐shout” and “human‐talk” category for modeling the probability of fleeing, and the natural predator treatment into a “wolf” and “cougar” category for modeling the proportion of time alert.

To model the probability of fleeing from the deterrent, we used a mixed‐effect logistic regression model using the variable, “Fled”, as the response variable (Table 2). To explore behavioral changes of interest, we grouped behaviors into “alert” and “relaxed” categories. Vigilance, running, and out of frame were grouped as “alert” behaviors and all other recorded behaviors (walking, standing, feeding, and investigating) were grouped as “relaxed” behaviors (Table 2). We included the time spent out of frame during and after each triggering event in the alert category as a measure of deterrence caused by the stimulus (Bhardwaj et al. 2022). All recorded behaviors were mutually exclusive so that the proportion of time spent relaxed and alert in each event summed to one. To determine how the alert time changed as a result of the various stimuli, we used a mixed‐effect logistic regression model and built a separate model for the proportion of time spent alert in each time period (before, during, and after stimulus) and each species. This approach allowed us to characterize behavioral responses during each distinct phase of the trial independently, while also reducing statistical complexity by limiting the number of interaction terms required.

We conducted all analyses using the “glmmTMB” function (glmmTMB version 1.1.9) in R. Differences in animal responses between sites were accounted for by fitting random intercepts for each site. In our models, the intercept represented the reference category of a single adult female being exposed to the bird stimulus without lights during daytime on the first day of the experiment. We standardized numeric variables by mean‐centering (mean = 0) and scaling (SD = 1; Hosmer and Lemeshow 2000). As a first step for identifying relevant predictor variables, we ran univariate generalized linear mixed models (GLMMs) and eliminated predictors associated with *p* > 0.25 from further analyses (Hosmer and Lemeshow 2000). With the retained variables, we used AIC corrected for small sample sizes (AICc; Akaike 1998; Burnham and Anderson 2002) to select models from all subsets and considered models within ΔAICc ≤ 2 to be top main effects models. We defined habituation as a decline in fleeing or alert behavior in response to the deterrent over time. To determine if elk and deer showed signs of habituation towards specific stimuli, we tested for a 2‐way interaction between each of audio type and trial day, and light stimulus and trial day. Additionally, we tested for a 2‐way interaction between light stimulus and audio type, and between a site's initial treatment (audio‐only or audio‐light) and trial day. We added each interaction term independently to the top main effects model. We re‐ran all subsets to identify a new set of top models. We retained models within ΔAICc ≤ 2 of the model with the lowest AICc value and present *p*‐values to maximize information for readers (Symonds and Moussalli 2011; Murtaugh 2014).

#### 2.5.2 Site‐Specific Variation in Habituation

To measure variation in the rate of habituation to stimuli among sites, we used a mixed effect logistic regression model for each species and included a random slope for trial day by site. The response variable was the proportion of time spent alert during and after exposure to the stimuli. We selected the most parsimonious random slope model for each species based on AICc (Akaike 1998; Burnham and Anderson 2002) and extracted the random slopes using the “ranef” function from the lme4 package in R. These random slopes represent the degree to which individual sites deviated from the overall (population‐level) rate of habituation. A more negative slope indicates a faster decline in alert behavior over time at that site, reflecting a faster rate of habituation relative to the population average.

To determine whether site characteristics influenced the rate of habituation to stimuli at each of 14 study sites for elk and 15 study sites for deer, we used the extracted random slopes as the response variable in multiple linear regression analyses using the lm function in R. Only 14 sites were used for elk because one site did not receive elk visits during our study. Our predictor variables included four landscape variables describing human infrastructure and two variables describing the intensity of animal use (Table S1). We used an all‐subsets modeling approach following the procedure described above for predicting the probability of fleeing and the proportion of time alert. We assessed collinearity using Pearson's correlation values (Dormann et al. 2013) and removed the lower performance predictor for correlated pairs (*r* > 0.6). We tested for a two‐way interaction between house density and distance to highway by adding it to the top main effects model. We considered models with the lowest AICc values (ΔAICc ≤ 2) to be the best descriptors of the site‐specific rate of habituation.

## 3 Results

We collected 987 events of ungulates adjacent to our deterrent devices, but we removed events where the deterrent failed to produce sound and light when triggered (*n* = 87), that were too dark to analyze (*n* = 7), or when the individual left the field of view within the first 8 s (before the stimulus occurred) and did not return (*n* = 125). Additionally, to minimize pseudo replication, we removed events in which the focal individual did not leave and triggered the deterrent again after the 1‐min delay (elk: *n* = 69; deer: *n* = 19). The final dataset included 680 ungulate events: 368 of elk and 312 of deer. The average group size was three for elk (range 1–26) and one for deer (range 1–5). The majority of ungulates analyzed were adult females (elk: *n* = 209; deer: *n* = 163) followed by adult males (elk: *n* = 100; deer: *n* = 57), juveniles (elk: *n* = 40; deer: *n* = 41), and unknown adults (elk: *n* = 19; deer: *n* = 51). Of the elk events, 168 occurred during the audio‐light treatment and 200 occurred during the audio‐only treatment. For the deer events, 135 events occurred during the audio‐light treatments and 177 events occurred during the audio‐only treatments.

### 3.1 Probability of Fleeing

**FIGURE 3:**
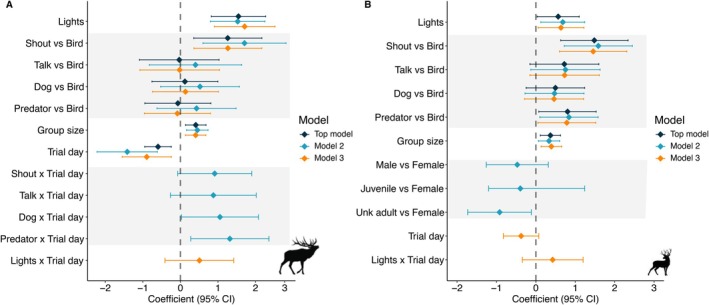
Coefficient estimates with 95% confidence intervals for the top models from logistic regression predicting the likelihood of elk (A) and deer (B) fleeing from the site in response to acoustic and visual stimuli triggered by remote cameras on the edges of crop fields in the Cowichan Valley, BC. Colors distinguish top candidate models based on AICc ranking. Gray shaded areas group terms belonging to audio type and sex and age class variables.

Across all stimulus types, elk fled following 19% of the events while deer fled following 43% of the events. In the majority of cases, individuals fled during the playback (elk = 90%; deer = 86%) rather than after it. Elk fled 36% of the time when exposed to human shouts (*n* = 53), more often than all the other categories (*p* ≤ 0.019 for all comparisons) with no differences among talking (13%; *n* = 53), natural predator (16%; *n* = 83), dog (16%, *n* = 88), or bird (17.5%; *n* = 91; *p* ≥ 0.68 for each; Figure 3; Figure S1; Table S3). Deer fled most often in response to human shouts (64% of the time; *n* = 45), followed by talking (46%; *n* = 39) and natural predators (45%; *n* = 75). Fleeing was more frequent in response to shouts (OR = 4.4, 95% CI [1.88, 10.33], *p* < 0.001), natural predators (OR = 2.24, 95% CI [1.08, 4.64], *p* = 0.031), talking (OR = 2.05, 95% CI [0.86, 4.93], *p* = 0.10), and dogs (OR = 1.64, 95% CI [0.78, 3.45], *p* = 0.19) compared to birds, with the latter two having confidence intervals that overlap one (Figure 3; Figure S1; Table S3).

The all subsets modeling approach resulted in three models within Δ2 AICc predicting the probability of fleeing for each of elk and deer (Table 3). The largest effect was caused by light; when lights were activated, elk were twice as likely to flee (26%) than when they were not activated (13%; Figure 3; Figure S1). Additionally, both species were more likely to flee in larger groups (elk: OR = 1.51, 95% CI [1.15, 1.98], *p* = 0.0032; deer: OR = 1.45, 95% CI [1.12, 1.87], *p* = 0.0047; Figure 3). The likelihood of fleeing did not differ between females, males, and juveniles for either species (*p* ≥ 0.24 for each; Figure 3; Figure S1). Deer that could not be identified as male or female (“unknown” sex) fled less than adult female deer (OR = 0.40, 95% CI [0.18, 0.89], *p* = 0.025; Figure 3; Figure S1). Additionally, the probability of fleeing did not differ depending on whether exposure was during the day or night (elk: OR = 1.1, 95% CI [0.52, 2.37], *p* = 0.79; deer: OR = 0.89, 95% CI [0.51, 1.57], *p* = 0.70). Elk were less likely to flee with increasing trial day (OR = 0.55, 95% CI [0.39, 0.78], *p* < 0.001), but deer were not (OR = 0.94, 95% CI [0.73, 1.22], *p* = 0.66; Figure 3). Sites that deployed both audio and light in the first half of the experiment did not exhibit a greater reduction in fleeing behavior over time compared to sites that deployed audio only in the first half (elk: OR = 1.06, 95% CI [0.43, 2.65], *p* = 0.89; deer: OR = 0.89, 95% CI [0.51, 1.52], *p* = 0.65). A weak positive interaction between light stimulus and trial day was present in our top models indicating that the probability of fleeing by both species was somewhat higher with increasing trial day when the lights were activated (elk: OR = 1.66, 95% CI [0.66, 4.16], *p* = 0.28; deer: OR = 1.53, 95% CI [0.71, 3.31], *p* = 0.28; Table 3; Figure 3). Additionally, we found evidence suggesting that elk were more likely to flee with increasing trial day when exposed to natural predator audio (OR = 3.73, 95% CI [1.32, 10.57], *p* = 0.013), dog audio (OR = 2.87, 95% CI [1.03, 8.0], *p* = 0.044), human shouts (OR = 2.49, 95% CI [0.93, 6.68], *p* = 0.069), and humans talking (OR = 2.41, 95% CI [0.77, 7.56], *p* = 0.13) compared to bird audio (Table 3; Figure 3).

**TABLE 3:** Model selection table for top candidate logistic regression models (i.e., models within 2 ΔAICc from the top model) explaining the probability of fleeing from the deterrent for elk and deer in the Cowichan Valley, BC.

Model #	Model terms	Log lik	df	AICc	ΔAICc	AICc weight
**Elk**						
1	Lights + audio + trial day + group size	−150.9	9	320.4	0.00	0.43
2	Lights + audio + trial day + group size + audio × trial day	−147.0	13	321.1	0.65	0.31
3	Lights + audio + trial day + group size + lights × trial day	−150.4	10	321.3	0.92	0.27
**Deer**						
1	Lights + audio + group size	−192.3	8	401.1	0.00	0.44
2	Lights + audio + group size + sex age	−189.4	11	40.6	0.49	0.34
3	Lights + audio + group size + trial day + lights × trial day	−109.9	10	402.5	1.41	0.22

*Note:* Model # corresponds to the model identifiers used in Figure 3 to distinguish between top models. All models include site as a random intercept.

### 3.2 Alert Behaviors

**FIGURE 4:**
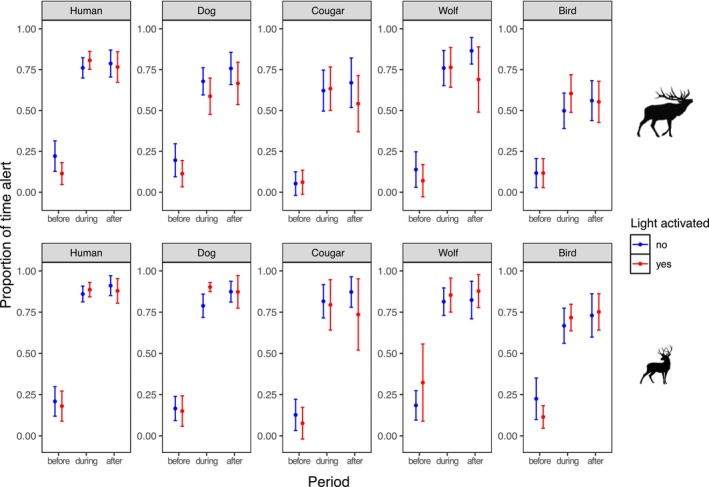
Average proportion of time spent alert for elk (top panels) and deer (bottom panels) before, during, and after exposure to acoustic and visual stimuli on crop fields in the Cowichan Valley, BC. Panels represent the different acoustic treatments and colors represent whether the lights were activated (red) or not (blue). The “Human” treatment includes vocalizations of humans shouting and humans talking in a conversational tone. Error bars represent standard deviations.

Before exposure to stimuli, the average time spent alert was 13% for elk and 18% for deer (Figure 4). Across treatments, alert time increased during exposure to 67% for elk and 81% for deer, and remained high after exposure at 69% and 84%, respectively (Figure 4; Figure S2). In events where individuals did not flee and stayed in frame, the focal individual remained alert for the entire duration of the post‐playback period 39% of the time for elk and 54% for deer. During exposure, elk spent the most time alert when hearing humans (78% of the time) followed by wolves (76%), dogs (64%), cougars (63%), and birds (55%). When exposure ended, elk remained more alert after hearing humans (OR = 2.73, 95% CI [1.44, 5.18], *p* = 0.0021), dogs (OR = 2.21, 95% CI [1.16, 4.22], *p* = 0.016), and wolves (OR = 3.0, 95% CI [1.18, 7.57], *p* = 0.021) than birds, with intermediate responses to cougars (OR = 1.11, 95% CI [0.53, 2.37], *p* = 0.77; Table S4). Deer spent the most time alert when hearing humans (87%) followed by dogs (83%), wolves (83%), cougars (81%), and birds (69%). When exposure ended, deer remained more alert after hearing humans (OR = 2.99, 95% CI [1.25, 7.13], *p* = 0.014) and dogs (OR = 2.41, 95% CI [1.04, 5.61], *p* = 0.041) than birds, but not after hearing wolves (OR = 1.85, 95% CI [0.70, 4.86], *p* = 0.21) or cougars (OR = 1.60, 95% CI [0.56, 4.53], *p* = 0.38; Table S4).

**FIGURE 5:**
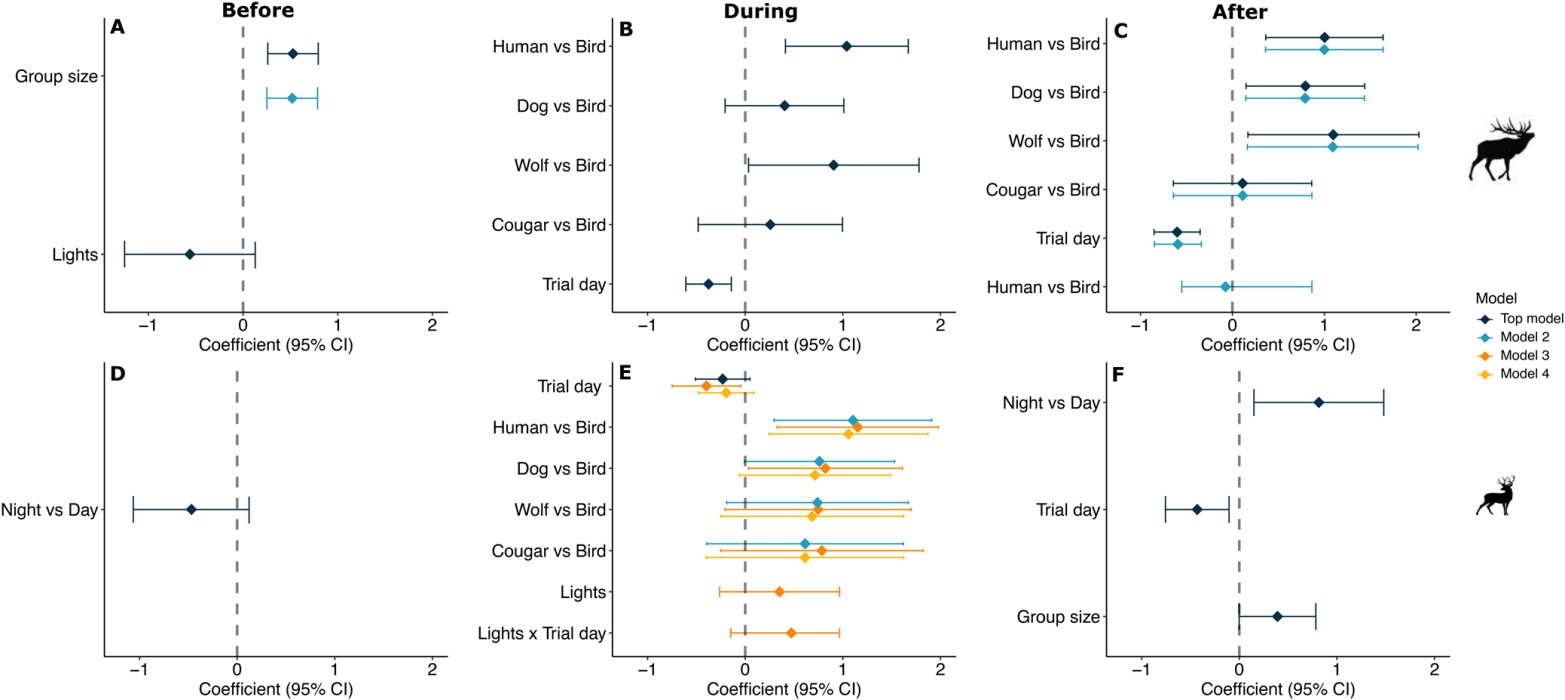
Coefficient estimates with 95% confidence intervals for the top models from logistic regression predicting the likelihood of elk (A–C) and deer (D–F) being alert before, during, and after exposure to acoustic and visual stimuli triggered by remote cameras on the edges of crop fields in the Cowichan Valley, BC. The “Human” treatment includes vocalizations of humans shouting and humans talking in a conversational tone. Colors distinguish top candidate models based on AICc ranking.

The addition of light did not affect alert time during or after exposure to the stimulus for either species (*p* ≥ 0.42 for all comparisons; Figure 4). Sex and age class were not present in the top models for either species (Table 4). Diel period was present in the top models for deer but not elk, suggesting that deer tended to remain more alert after exposure when the event was at night (OR = 2.26, 95% CI [1.16, 4.40], *p* = 0.016; Table 4; Figure 5). Group size had no effect on alert time during and after exposure to stimuli for either species, but elk were more likely to be alert in larger groups before exposure to stimuli occurred (OR = 1.69, 95% CI [1.30, 2.21], *p* ≤ 0.001; Figure 5). Across all treatment types, both species were less alert after exposure to stimuli with increasing trial day (elk: OR = 0.55, 95% CI [0.43, 0.70], *p* ≤ 0.001; deer: OR = 0.65, 95% CI [0.47, 0.90], *p* = 0.009; Figure 5). Sites that deployed both audio and light in the first half of the experiment did not exhibit a greater reduction in alert behavior over time compared to sites that deployed audio only in the first half (elk: OR = 1.17, 95% CI [0.73, 1.88], *p* = 0.46; deer: OR = 1.06, 95% CI [0.59, 1.91], *p* = 0.84). Additionally, the interaction between light stimulus and trial day was present in our top models for predicting the probability of deer being alert during exposure (OR = 1.60, 95% CI [0.86, 3.0], *p* = 0.14), indicating that deer tended to remain more alert with increasing trial day when the lights were activated (Table 4; Figure 5).

**TABLE 4:** Model selection table for top candidate logistic regression models (i.e., models within 2 ΔAICc from the top model) explaining the proportion of time elk and deer spent alert in response to the stimuli in the Cowichan Valley, BC.

Model #	Model terms	Log lik	df	AICc	ΔAICc	AICc weight
**Elk**						
*Before*						
1	Group size + lights	−134.0	4	276.0	0.00	0.57
2	Group size	−135.3	3	276.0	0.56	0.43
*During*						
1	Audio + trial day	−221.5	7	457.2	0.00	1.0
*After*						
1	Audio + trial day	−208.4	7	431.0	0.00	0.73
2	Audio + trial day + lights	−208.3	8	433.0	1.99	0.27
**Deer**						
*Before*						
1	Diel period	−143.4	3	292.9	0.00	0.54
2	Null model	−144.6	2	293.3	0.32	0.46
*During*						
1	Trial day	−152.1	3	310.3	0.00	0.26
2	Audio	−149.3	6	310.8	0.50	0.20
	Null model	−153.4	2	310.9	0.61	0.19
3	Audio + lights + trial day + lights × trial day	−146.1	9	310.9	0.61	0.19
4	Audio + trial day	−148.4	7	311.1	0.81	0.17
*After*						
1	Diel period + trial day + group size	−132.0	5	274.1	0.00	1.0

*Note:* Model # corresponds to the model identifiers used in Figure 5 to distinguish between top models. All models include site as a random intercept.

### 3.3 Variation in Habituation

**FIGURE 6:**
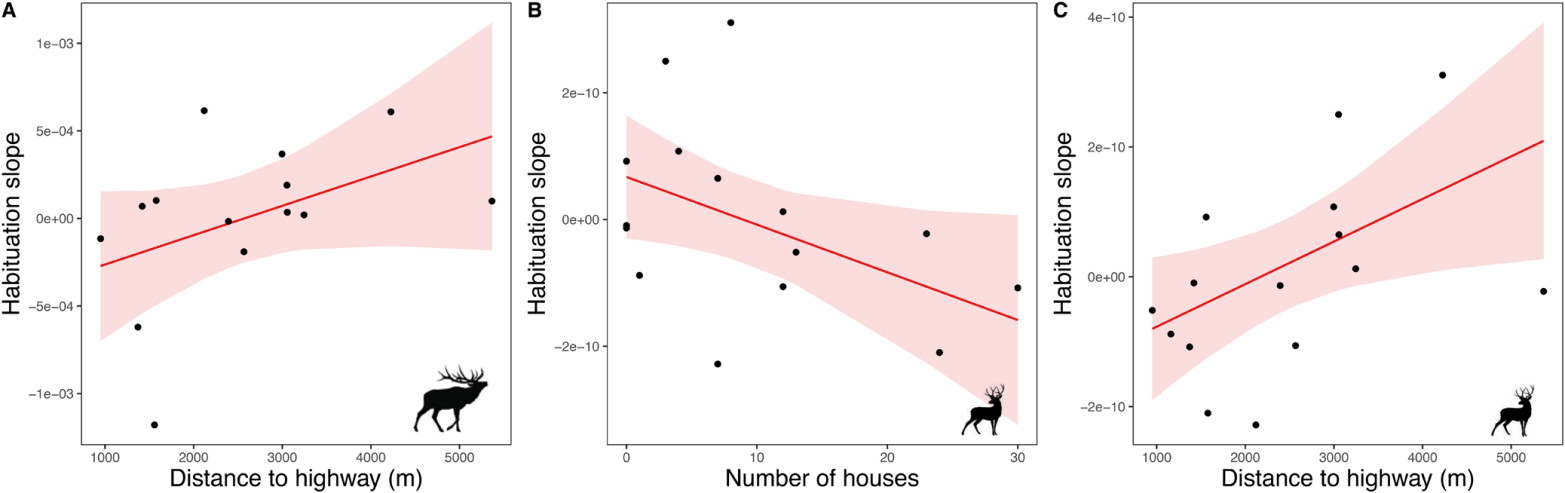
The estimated habituation slope with distance to the highway (m) for elk (A) and deer (C) and with the number of houses within a 500 m radius of the camera site for deer (B) in the Cowichan Valley, BC. The habituation slopes represent the change in alertness to acoustic and visual stimuli over the 45‐day study period. The lines reflect the model estimated habituation slope, and the shaded areas display 95% confidence intervals.

The most parsimonious random slope model for elk included trial day and group size (AICc = 897.2; AICc weight = 0.70) and, for deer, trial day, time of day, and period (AICc = 588.5; AICc weight = 1.0). The predicted probability of alertness for elk declined from 86.4% on day 1 to 55.0% on day 45, representing a 36.3% relative decrease in alertness over the course of the trial period. For deer, the predicted probability of alertness declined from 84.8% on day 1 to 62.9% on day 45, representing a 25.8% relative decrease in alertness. For elk, site‐specific random slopes ranged from −1.2 × 10^−3^ to 6.2 × 10^−4^, while for deer they ranged from −2.8 × 10^−10^ to 3.1 × 10^−10^. In the model describing the rate of habituation for elk, the null model with only the intercept was the best model. A second model with a ΔAICc of 0.27 suggested a weak relationship between elk habituation and proximity to the highway, where elk tended to have a faster rate of habituation (more negative habituation slopes) at sites closer to the highway (β = 2.0 × 10^−4^; *t* = 1.7; *p* = 0.11; Figure 6). For deer, the number of houses and distance to the highway were present in the top model, showing that deer had a faster rate of habituation at sites with more houses (β = −7.2 × 10^−11^; *t* = −2.2; *p* = 0.046) and a slower rate of habituation at sites further from the highway (β = 8.1 × 10^−11^; *t* = 2.5; *p* = 0.028; Figure 6). Neither the number of events nor the average group size at a site emerged in the top model set for elk or deer.

## 4 Discussion

Human‐wildlife conflict around the world frequently involves ungulate depredation of crops, which might be mitigated with the use of motion‐activated deterrents. We used remote cameras to activate deterrents on crop fields and compared the responses of elk and deer to various acoustic stimuli with and without the presence of flashing LED lights. We found that light increased the flight responses, but not alert time, of both species across acoustic stimuli, and the sound of human shouts tended to elicit greater flight responses than recordings of humans talking, natural predators, dogs, and bird sounds. Responses declined with trial day for both species, but occurred more rapidly for elk than for deer, and less rapidly when deterrents included flashing lights. Deer tended to habituate more quickly at sites with a higher density of houses and closer to highways, but the effects of landscape factors on the rate of elk habituation were not apparent.

Our result shows that both species were most reactive to human shouts, resulting in a flight response 36% of the time for elk and 64% of the time for deer. This is similar to other studies in which wildlife responded more strongly to human vocalizations than to those of natural predators (Clinchy et al. 2016; Smith et al. 2017; Epperly et al. 2021; Crawford et al. 2022; Widén et al. 2022). However, the majority of these other studies used recordings of humans talking in a conversational tone, which we found to produce a lesser response than for humans shouting. In our study, neither species was more likely to flee in response to humans talking than natural predators or dogs. In fact, elk were no more likely to flee from human conversation than they were from bird sounds, suggesting they were quite habituated to human presence on the landscape. Other studies on elk ( *Cervus elaphus* ; Cassirer et al. 1992) and Svalbard reindeer ( *Rangifer tarandus platyrhynchus* ; Colman et al. 2001) have shown that flight responses to human cues tend to be lesser where benign interactions with humans occur frequently, relative to areas where there is limited human interaction. The lesser response to recordings of human conversation in our study may have occurred because it was conducted in a landscape with a high degree of human activity, tourism, and infrastructure, where frequent benign encounters between humans and elk are likely common. Additionally, the increased reactiveness to shouts suggests that ungulates may be able to perceive the level of threat conveyed in the broadcast of a human vocalization and that shouting may be a stronger fear cue than conversational tones, especially in areas like ours where conflict with elk is common and shouting may accompany other forms of deterrence, like chasing or throwing projectiles.

We were surprised by the similarity in alert responses by both species to sounds of natural predators and bird vocalizations. While both species were least alert in response to bird stimuli, the time spent alert was not different when hearing cougars for either species or when hearing wolves for deer. However, elk in our study exhibited the most rapid decline in flight responses to bird audio (Figure 3), suggesting that the stimulus was quickly perceived as non‐threatening. In a similar study conducted on moose at saltlick stones, researchers found that reactions to bird vocalizations were stronger than expected and that moose exposed to bird stimuli spent more time vigilant compared with silent controls and an equal time being vigilant when exposed to human stimuli (Bhardwaj et al. 2022). We did not use bird alarm calls, but the sudden onset of the bird broadcast may have elicited surprise in our study and others (Brown et al. 2015; Bhardwaj et al. 2022), and we may not have adequately standardized other characteristics, such as pitch, frequency, and amplitude (Clark and Dunn 2022). Additionally, wild animals may not recognize acoustic recordings as representing the predators researchers intend to emulate (McGregor 2000), and it is worth noting that wolves and cougars rarely use acoustic signals when hunting. Limitations in speaker quality and electronic noise from the system may have further prevented the playbacks from accurately simulating the true, informative wavelengths of natural calls and human voices (Bhardwaj et al. 2022). As a result, responses might not fully reflect ecologically relevant prey responses.

Our models predicted that the addition of lights increased the odds of fleeing by 4.7 in elk and 1.8 in deer, supporting the findings by others that engaging multiple sensory modalities increases animal responsiveness (Invertebrates: Uetz et al. 2009; Fish: Lukas et al. 2021; Birds: Avery and Mason 1997; Mammals: Partan et al. 2009). Multimodal deterrents may reduce ambiguity by providing multiple, reinforcing cues that signal danger (Conover 2002; Munoz and Blumstein 2012). In terrestrial mammals, sudden flashes of light may act as a startle cue or be perceived as a sign of proximity of humans or predators (Koehler et al. 1990). This convergence of sensory information may allow for faster and more accurate assessments of risk, promoting stronger anti‐predator responses in wildlife (Bomford and O'Brien 1990; Elmer et al. 2021). In contrast to the pronounced effect of lights on flight responses, lights did not alter alert time. One potential reason for this effect is that the flashing lights allowed elk to better locate the source of the sound and the cessation of the stimulus a few seconds later. Without the light cue, elk may have spent more time alert searching for the potential threat. Future studies on the combined effect of cues as well as the duration of cues on ungulate behavior will be important for the continued development of nonlethal management tools (Ramirez et al. 2024).

We did not find an effect of age or sex class on anti‐predator responses with one exception. Deer that we could not identify as male or female, usually because it was too dark and/or they were not close enough to discern the presence of antlers, fled less often than adult females. As diel period did not affect the probability of fleeing, it is possible that “unknown” deer fled less than adult females because they were usually further away from the deterrent and thus the intensity of the stimulus was reduced (Bomford and O'Brien 1990). Additionally, group size had no effect on alert time, but both species were more likely to flee in larger groups, a pattern also observed in roe deer ( *Capreolus capreolus* ; De Boer et al. 2004), fallow deer ( *Dama dama* ; De Boer et al. 2004), and caribou ( *Rangifer tarandus* ; Aastrup 2000). These findings contradict the hypothesis that individuals may have greater perceptions of safety in larger groups and are therefore less reactive to threats (dilution effect; Stankowich and Coss 2006). However, larger ungulate groups can be expected to have greater flight responses because they are more likely to contain a particularly reactive or wary individual, whose movements influence the rest of the group (Stankowich 2008). In our study, when one individual decided to flee, the entire group followed, suggesting that group responses matched the more reactive individuals (Found and St. Clair 2018; Found 2019).

Elk reduced their flight responses, and both species reduced their alert responses with increasing trial day, demonstrating a clear effect of habituation to the deterrents over time. We found some evidence that the addition of lights reduced the rate of habituation for both species. While these relationships were not strong, they suggest that multimodal stimuli may reduce the rate of habituation to simulated predator cues, similar to findings in ring‐billed gulls ( *Larus delawarensis* ; Lecker et al. 2015). Multimodal stimuli may generally be more reliable to animals, thereby slowing the rate with which they habituate to them (Blumstein 2016). These findings reinforce the importance of integrating multiple sensory cues in the design of wildlife deterrents, not only to elicit stronger immediate responses (above) but also to potentially mitigate the rapid onset of habituation by sustaining the perceived relevance of the threat (Conover 2002; Munoz and Blumstein 2012).

Our study included a unique approach for studying the process of habituation, which is a perennial topic of interest for behavioral studies in both basic contexts (Groves and Thompson 1970; Rankin et al. 2009) and applied ones (Thompson and Henderson 1998; Uchida and Blumstein 2021; Ramirez et al. 2024). We measured site‐specific rates of habituation in our study area by extracting random slopes for the relationship between alert time and trial day over the experimental period. While the overall variation among sites was small, these random effects allowed us to compare the relative speed of habituation across locations and test whether this variation was associated with local landscape characteristics. The most parsimonious model showed that deer habituated more quickly at sites surrounded by more houses and at sites closer to the highway. For elk, despite a model with distance to the highway being in the top model set, the most parsimonious model was the null model, suggesting that the effect of human infrastructure on the rate of elk habituation was not apparent. At least for deer, these results suggest that exposure to humans can drive variation in the rate of habituation to acoustic and visual deterrents. Ungulates that use human activity as a protective buffer against the risk of predation may habituate to human‐related disturbances more quickly (i.e., predator shield; Berger 2007). Desensitization to deterrents involving human cues may be accelerated in areas where individuals have already developed a higher tolerance for human presence and associated disturbances, and the scale at which this operates may be species‐dependent. Deterrents might challenge this habituation process with greater variation in stimuli types (Blumstein 2016) or by pairing stimuli with a real threat (Babińska‐Werka et al. 2015).

Our study had several limitations that may reduce the generality of our findings. We used a small number of sites (*n* = 15) and a large number of stimuli combinations (*n* = 10), causing low statistical power among acoustic treatments (Table S2). The generally low responsiveness of elk to our deterrents also limited our ability to measure the effects of landscape context on rates of habituation. Moreover, our distribution of sites might not have captured enough variation in landscape‐level human disturbance to identify these effects (Stankowich 2008). A second limitation is that the onset of an archery‐only hunting season on September 1st, during our 6‐week study, may have increased the propensity for elk to ignore our recorded stimuli relative to the danger they might perceive from hunters in more remote locations. A third limitation is the short time period between playbacks (1‐min minimum), which created the possibility of repeated exposures to the same individuals, dampening their responsiveness to stimuli. It is likely that this intensity of exposure contributed to the observed habituation over the course of the experiment (Bomford and O'Brien 1990; Winslow et al. 2002; Bhardwaj et al. 2022). Additionally, the deterrent used in our study simulated threats through sound and light but imposed no real consequence, which may have also contributed to the rapid habituation over time. This reflects a common limitation with fear‐based deterrents: without an associated cost, animals may quickly learn that the stimuli pose no real risk, reducing long‐term effectiveness (Groves and Thompson 1970; Blumstein 2016). Finally, our unmarked population made it impossible to identify variation in responses among individuals (Found and St. Clair 2018), which might be corrected in future studies on marked populations (Blumstein 2016).

## 5 Conclusion

Animal‐activated deterrents are among the tools that could reduce the need for lethal management of wildlife to minimize conflict and increase coexistence (Price et al. 2022; Ramirez et al. 2024). Our findings have practical implications for the design and deployment of deterrents in agricultural landscapes. Specifically, our study supports past evidence that aggressive vocalizations by humans induce a fear response in ungulates, engaging multiple sensory modalities increases the efficacy of deterrents and slows the rate of habituation to them, and most novelly, that random effects can provide a measure of site‐based variation in habituation in populations of unmarked animals. Differences between elk and deer in our study suggest that elk are especially prone to habituation, consistent with several other studies (Thompson and Henderson 1998; Found and St. Clair 2016). In practice, this means that stationary deterrents may be more effective for short‐term exclusion or in areas where elk are not yet highly habituated to human activity. Managers may need to rotate stimuli or pair deterrents with additional reinforcement to sustain effectiveness over time. We focused on the relative strength of short‐term risk effects in response to different audio‐visual signals at small spatial scales, but whether these behavioral changes translate to fitness consequences or shifts in resource use that could scale up to population‐level effects cannot be determined by this study, but would be an interesting avenue of future research (DeWitt et al. 2019). Additionally, future studies should investigate the combinations of stimuli and spatial characteristics of the landscape that influence habituation rates to animal‐activated deterrents, ideally with robust sample sizes and marked animals. Increasing our understanding of what drives variation in habituation among and within target species will support more effective management of wildlife populations at relevant spatial and temporal scales.

## Author Contributions

**Kate L. Rutherford:** conceptualization (equal), data curation (lead), formal analysis (lead), funding acquisition (supporting), investigation (equal), methodology (lead), project administration (lead), visualization (lead), writing – original draft (lead), writing – review and editing (equal). **Colleen Cassady St. Clair:** conceptualization (equal), formal analysis (supporting), funding acquisition (equal), methodology (supporting), resources (equal), supervision (equal), visualization (supporting), writing – review and editing (equal). **Darcy R. Visscher:** conceptualization (lead), formal analysis (supporting), funding acquisition (equal), methodology (supporting), resources (equal), supervision (equal), visualization (supporting), writing – review and editing (supporting).

## Conflicts of Interest

The authors declare no conflicts of interest.

## Supporting information


Appendix S1

Appendix S2

## Data Availability

Data are accessible as Appendix S1.
