# Supplementary material for: Elk and Deer Habituate to Stationary Deterrents in an Agricultural Landscape
Source: Ecol Evol. 2025 Jul 10;15(7):e71752. doi: 10.1002/ece3.71752 (PMC12243069; doi:10.1002/ece3.71752)
Supplement: Supplementary file 2 — Appendix S2 [file ECE3-15-e71752-s002.docx]

**Supplementary file**

**Table S1.** List of 6 predictor variables classified under 2 categories that are considered in the site-specific variation in habituation analysis for elk and deer in the Cowichan Valley, BC.

| Group | Covariates | Description | Source | Abbreviation |
| --- | --- | --- | --- | --- |
| Landscape-level human presence | Houses | Number of addresses in the 500m radius around the camera site | QGIS | Houses |
|  | Road density | Total length of roads (m) within the 500m radius around the camera site | QGIS | Road_density |
|  | Distance to highway | Shortest distance from camera site to major highway | QGIS | Dist_highway |
|  | Proportion of cropland | Proportion of cropland in the 500m radius around the camera site | QGIS | Crop_proportion |
| Intensity of use | Number of events | The number of events of the focal species at each site | Camera trap data | Num_event |
|  | Average group size | The average group size of the focal species at each site | Camera trap data | Avg_groupsize |

**Table S2.** Results of a Chi-squared and ANOVA test to determine whether the probability of fleeing or being alert differed in response to stimuli presented on crop fields in the Cowichan Valley, BC, within the groups of 3 birds, 2 natural predators, and 2 types of human sounds. A Chi-squared test was conducted to determine differences in the probability of fleeing and an ANOVA was conducted to determine differences in the proportion of time spent alert. Results are presented for elk and deer.

| Species | Audio category | Audio name | n | Fleeing | | Alert | |
| --- | --- | --- | --- | --- | --- | --- | --- |
|  |  |  |  | X^2^ | P-value | F value | P-value |
| Elk | Human | Talk | 53 | 6.17 | **0.013** | 1.43 | 0.23 |
|  |  | Shout | 53 |  |  |  |  |
|  | Natural predator | Wolf | 37 | 4.6e-32 | 1.0 | 5.98 | **0.015** |
|  |  | Cougar | 46 |  |  |  |  |
|  | Bird | Robin | 34 | 0.50 | 0.78 | 1.0 | 0.36 |
|  |  | Crow | 28 |  |  |  |  |
|  |  | Nighthawk | 29 |  |  |  |  |
| Deer | Human | Talk | 39 | 2.14 | 0.14 | 0.27 | 0.61 |
|  |  | Shout | 45 |  |  |  |  |
|  | Natural predator | Wolf | 43 | 0.22 | 0.64 | 0.97 | 0.33 |
|  |  | Cougar | 32 |  |  |  |  |
|  | Bird | Robin | 24 | 1.36 | 0.51 | 0.43 | 0.65 |
|  |  | Crow | 29 |  |  |  |  |
|  |  | Nighthawk | 22 |  |  |  |  |

**Table S3.** Pairwise comparisons of the probability that elk and deer would flee from a site after exposure to acoustic stimuli treatments on crop fields in the Cowichan Valley, BC. Pairwise comparisons were derived by changing the reference category in the top model based on AICc model selection.

| Comparison | Elk | | | Deer | | |
| --- | --- | --- | --- | --- | --- | --- |
|  | Estimate | Std. Error | P-value | Estimate | Std. Error | P-value |
| Shout - Bird | 1.26 | 0.46 | **0.0064** | 1.48 | 0.43 | **<0.001** |
| Talk - Bird | -0.030 | 0.54 | 0.96 | 0.72 | 0.45 | 0.10 |
| Natural predator - Bird | 0.067 | 0.45 | 0.88 | 0.80 | 0.37 | **0.031** |
| Dog - Bird | 0.12 | 0.45 | 0.79 | 0.50 | 0.38 | 0.19 |
| Talk - Shout | -1.29 | 0.55 | **0.019** | -0.76 | 0.49 | 0.12 |
| Natural predator - Shout | -1.33 | 0.47 | **0.0046** | -0.68 | 0.42 | 0.11 |
| Dog - Shout | ​​-1.14 | 0.46 | **0.014** | ​​​​​​-0.99 | 0.43 | **0.021** |
| Dog - Natural predator | 0.19 | 0.46 | 0.68 | -0.31 | 0.36 | ​​0.39 |
| Talk - Natural predator | -0.037 | 0.55 | 0.95 | -0.082 | 0.43 | 0.85 |
| Dog - Talk | 0.15 | 0.55 | 0.78 | -0.2 | 0.44 | 0.61 |

**Table S4.** Pairwise comparisons of the probability of being alert before, during, and after exposure to acoustic and visual stimuli on crop fields in the Cowichan Valley, BC. Pairwise comparisons were derived by changing the reference category in the top model based on AICc model selection. If audio treatment was not present in any of the top models **(**ΔAICc <2) then pairwise comparisons were taken from the univariate test. Results are presented for elk and deer.

| Comparison | Before | | During | | After | | |
| --- | --- | --- | --- | --- | --- | --- | --- |
|  | Estimate | P-value | Estimate | P-value | Estimate | P-value | |
| ***Elk*** | | | | | | | |
| Human - Bird | 0.39 | 0.36 | 1.04 | **0.0012** | 1.00 | **​​0.0021** | |
| Dog - Bird | 0.29 | ​​0.52 | 0.40 | 0.19 | 0.80 | **0.016** | |
| Wolf - Bird | -0.16 | 0.81 | 0.91 | **0.042** | 1.10 | **0.021** | |
| Cougar - Bird | -0.80 | 0.27 | 0.26 | 0.50 | 0.11 | 0.77 | |
| Dog - Human | -0.10 | 0.79 | -0.64 | 0.052 | -0.21 | 0.54 | |
| Wolf - Human | -0.55 | 0.36 | -0.13 | 0.77 | 0.09 | 0.85 | |
| Cougar - Human | -1.19 | 0.087 | -0.78 | **0.045** | ​​-0.89 | **0.024** | |
| Wolf - Dog | -0.44 | 0.47 | 0.50 | 0.27 | 0.30 | 0.53 | |
| Cougar - Dog | -1.09 | 0.13 | -0.15 | 0.70 | ​​-0.68 | ​​ 0.09 | |
| Cougar - Wolf | -0.65 | 0.44 | -0.65 | 0.19 | -0.99 | 0.060 | |
| ***Deer*** | | | | | | |  |
| Human - Bird | 0.21 | 0.62 | 1.10 | **​​0.0075** | 1.09 | **0.014** | |
| Dog - Bird | -0.03 | 0.94 | ​​0.76 | 0.052 | 0.88 | **0.041** | |
| Wolf - Bird | 0.42 | 0.39 | 0.74 | 0.12 | 0.61 | 0.22 | |
| Cougar - Bird | ​​-0.49 | 0.45 | 0.61 | ​​0.23 | 0.47 | 0.38 | |
| Dog - Human | -0.24 | 0.56 | -0.34 | 0.44 | -0.21 | 0.66 | |
| Wolf - Human | 0.21 | 0.65 | -0.36 | 0.48 | -0.48 | 0.38 | |
| Cougar - Human | -0.70 | 0.27 | -0.49 | 0.38 | -0.63 | 0.28 | |
| Wolf - Dog | 0.45 | 0.35 | -0.02 | 0.97 | -0.27 | ​​0.62 | |
| Cougar - Dog | -0.45 | 0.48 | -0.15 | 0.79 | -0.41 | 0.47 | |
| Cougar - Wolf | -0.90 | 0.18 | 0.13 | ​​0.83 | -0.14 | 0.82 | |

**
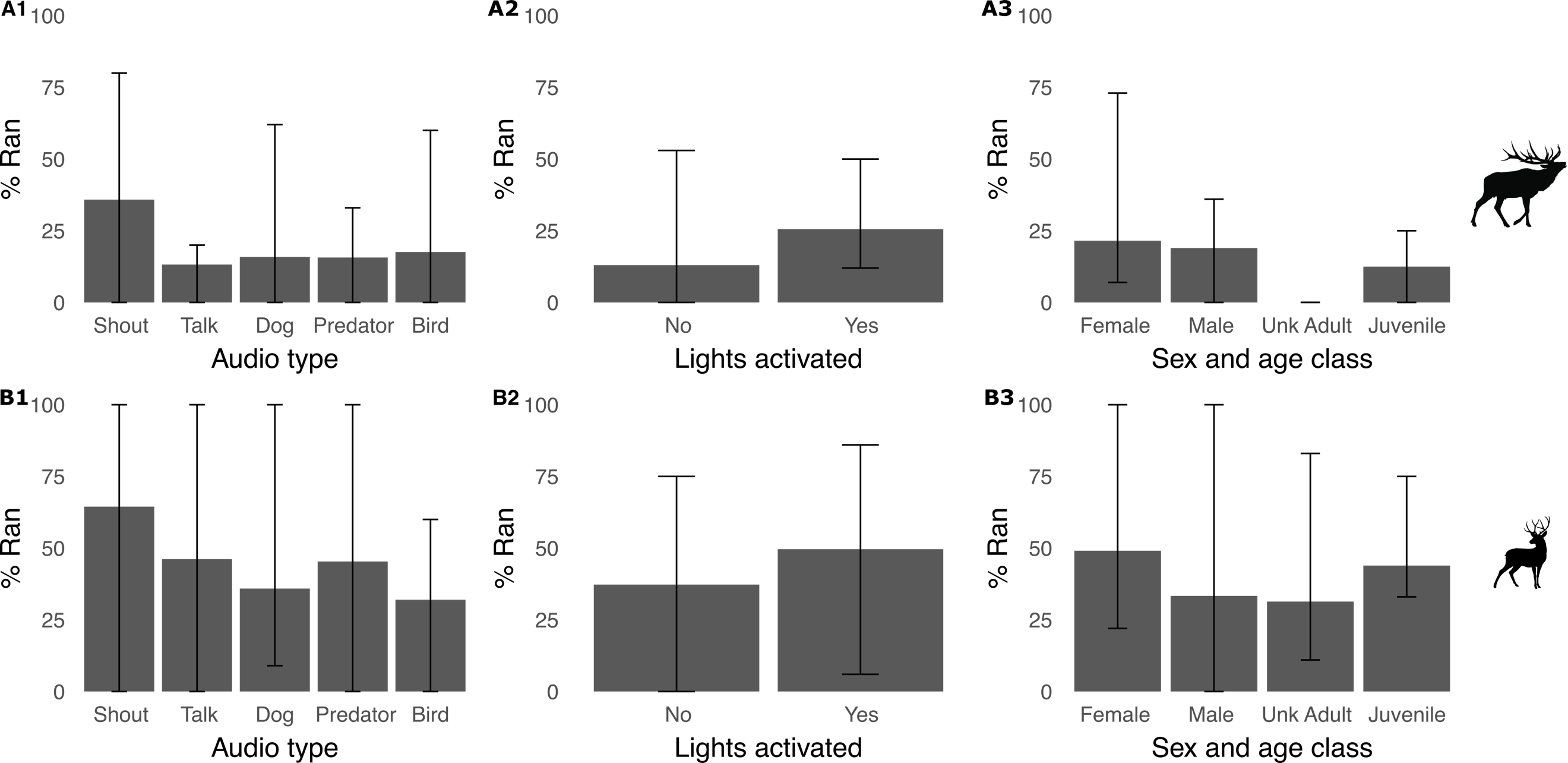
**

**Figure S1.** The percentage of time elk (A) and deer (B) fled in response to acoustic and visual stimuli on the edge of crop fields in the Cowichan Valley, BC. Panels show the percentage of time that each species ran to each audio treatment (1), light treatment (2) and by age and sex class (3). Error bars represent variation in the percentage ran by site for sites with at least 3 observations in each category.

**
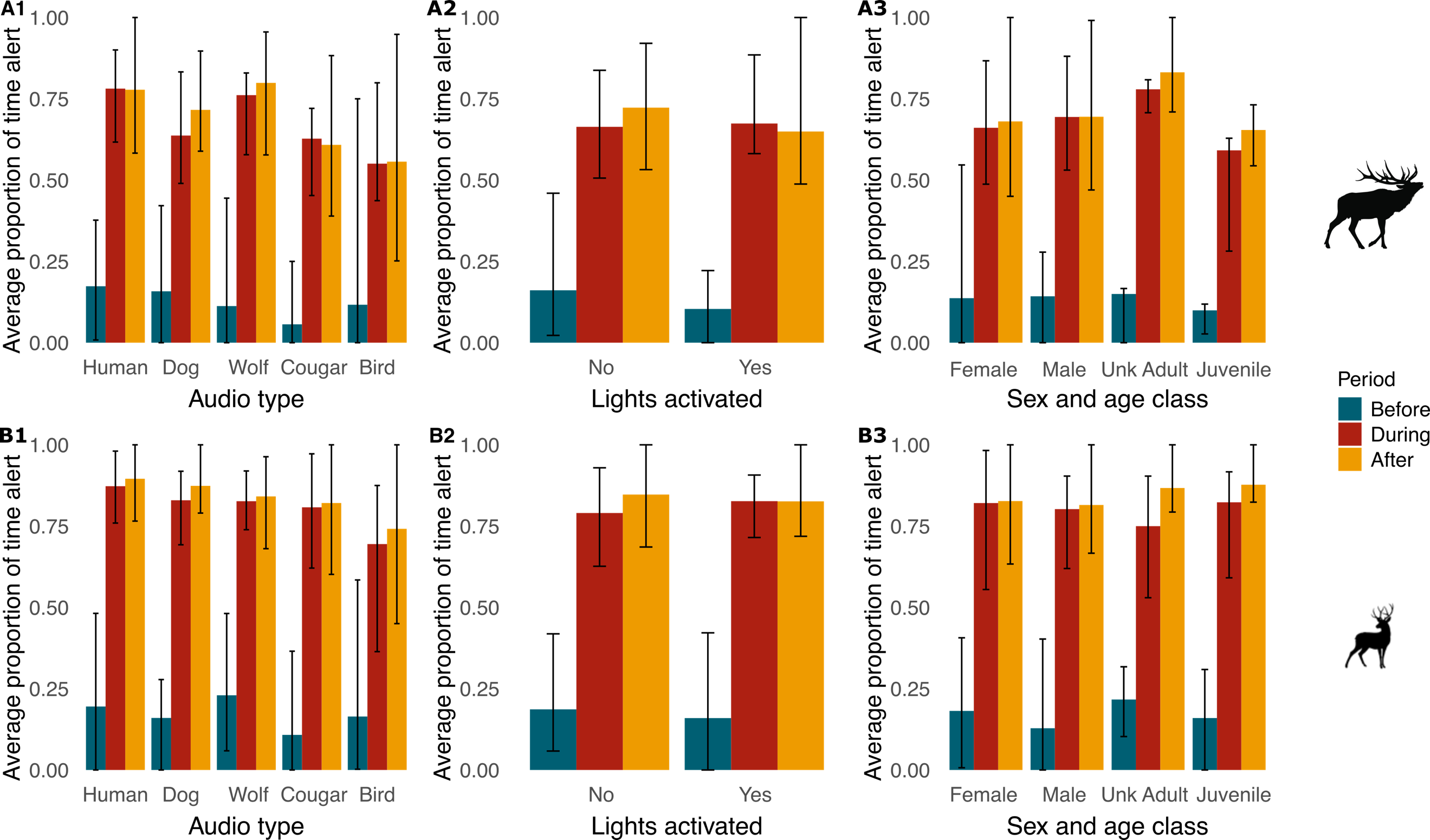
**

**Figure S2.** The average proportion of time elk (A) and deer (B) spent alert in response to acoustic and visual stimuli on the edge of crop fields in the Cowichan Valley, BC. Panels show the proportion of time that each species spent alert in response to each audio treatment (1), light treatment (2) and by age and sex class (3) before, during, and after exposure to stimuli. Error bars represent variation in the proportion of time spent alert by site for sites with at least 3 observations in each category.
